# Increased risk of fetal left–right asymmetry disorders associated with maternal SARS-CoV-2 infection during the first trimester

**DOI:** 10.1038/s41598-024-61778-w

**Published:** 2024-05-19

**Authors:** Yang Li, Yuemei Wang, Haifang Wu, Qi Li, Shizhen Li, Chunli Qiu, Shuo Qiu, Qingfang Niu, Xianmei Zhang, Yi Xiong, Guowei Tao

**Affiliations:** 1 Department of Medical Ultrasound, The First Affiliated Hospital of Shandong First Medical University & Shandong Provincial Qianfoshan Hospital, Shandong Medicine and Health Key Laboratory of Abdominal Medical Imaging, Jinan, China; 2 The Second Affiliated Hospital of Shandong First Medical University, Taian, 271000 China; 3 Jinan Maternal and Child Health Care Hospital, Jinan, China; 4 Qilu Hospital of Shandong University, No. 107, Wenhua West Road, Jinan City, 250012 China; 5 Linyi Maternal and Child Health Care Hospital, Shenzhen, China; 6 Luohu People’s Hospital, Third Affiliated Hospital of Shenzhen University, Shenzhen, China

**Keywords:** Left–right asymmetry disorders, SARS-CoV-2, Fetal, Ultrasound, Health occupations, Risk factors, Diseases, Infectious diseases, Reproductive disorders

## Abstract

Our center has observed a substantial increase in the detection rate of fetal left–right(LR) asymmetry disorders between March and May 2023. This finding has raised concerns because these pregnant women experienced the peak outbreak of SARS-CoV-2 in China during their first trimester. To explore the relationship between maternal SARS-CoV-2 infection and fetal LR asymmetry disorders. A retrospective collection of clinical and ultrasound data diagnosed as fetal LR asymmetry disorders was conducted from January 2018 to December 2023. The case–control study involved fetuses with LR asymmetry disorders and normal fetuses in a 1:1 ratio. We evaluated and compared the clinical and fetal ultrasound findings in pregnant women with SARS-CoV-2 infection and pregnant women without infection. The Student *t*-test was utilized to compare continuous variables, while the chi-squared test was employed for univariable analyses. The incidence rate of LR asymmetry disorders from 2018 to 2023 was as follows: 0.17‰, 0.63‰, 0.61‰, 0.57‰, 0.59‰, and 3.24‰, respectively. A total of 30 fetuses with LR asymmetry disorders and 30 normal fetuses were included. This case–control study found that SARS-CoV-2 infection (96.67% vs 3.33%, *P* = .026) and infection during the first trimester (96.55% vs 3.45%, *P* = .008) were identified as risk factors. The odds ratio values were 10.545 (95% CI 1.227, 90.662) and 13.067 (95% CI 1.467, 116.419) respectively. In cases of SARS-CoV-2 infection in the first trimester, the majority of infections (88.1%, 37/42) occurred between 5 and 6 weeks of gestation. We found that 43.7% (66/151) of fetuses with LR asymmetry disorder had associated malformations, 90.9% (60/66) exhibited cardiac malformations. SARS-CoV-2 infection during the first trimester significantly increases the risk of fetal LR asymmetry disorders, particularly when the infection occurs between 5 and 6 gestation weeks. The most common associated malformation is heart malformation.

## Introduction

The SARS-CoV-2 virus outbreak originated in December 2019 and was declared a global pandemic by the World Health Organization (WHO) in March 2020. On May 5, 2023, the WHO announced that the SARS-CoV-2 epidemic would no longer be considered a 'public health emergency of international concern'. However, the possibility of virus mutation still remains uncertain, and further observation is required to determine the long-term effects on the human body.

Pregnant women undergo physiological changes that affect their immune system, cardiovascular and respiratory systems, and maternal homeostasis^1,2^. These changes make them more susceptible to viral infections and increase the severity of respiratory diseases^3,4^. As a result, pregnant women are considered a high-risk group and are advised to take extra precautions during the SARS-CoV-2 pandemic.

The short- and long-term effects of maternal SARS-CoV-2 infection on the fetus are still unknown. Previous studies have reported adverse outcomes such as increased rates of preterm birth and newborn hospitalization in the NICU, as well as increased risk of adverse maternal outcomes^5–9^. Research on the impact of the virus on the fetus has mainly focused on placenta and fetal development^10–12^, with limited reports on whether it can cause fetal malformations^13^.

Human left–right (LR) asymmetry disorders can be classified into two categories: Situs Inversus and Heterotaxy. Situs inversus refers to a condition where the internal organs are arranged in a complete mirror image along the left–right axis. On the other hand, heterotaxy is characterized by an abnormal arrangement of the internal thoracoabdominal organs in relation to each other and the LR axis^14^. In 1600, Fabricius documented the first known instance of reversal of the liver and spleen in humans^15^. Later in 1897, Vehsemeyer was credited with demonstrating the transposition of the viscera through the use of X-ray imaging. Since then, imaging has become the preferred method for clarifying anatomy^16^.The incidence of situs inversus is approximately of 1:6000 to 1:8000 live births^17^, while the prevalence of heterotaxy has been estimated as 1 in 10,000 at birth^18^.

Our center observed that during 2023, the detection rate of fetal LR asymmetry disorders increased significantly, especially from March to May. This has raised concerns as these pregnant women coincided with the full outbreak of SARS-CoV-2 in China during their first trimester. We are currently investigating whether this increase is related to SARS-CoV-2. Due to the rarity of LR asymmetry disorders, gathering enough cases for study is challenging. Therefore, we have established a correlation between the two by utilizing data support from multiple centers. This study aimed to investigate the potential risk associated with SARS-CoV-2 infection and LR asymmetry disorders during pregnancy. We also considered the gestational age of infection, along with other variables. Furthermore, we evaluated and compared the clinical and fetal ultrasound findings in pregnant women with SARS-CoV-2 infection.

## Material and methods

### Study design and participants

**Figure 1:**
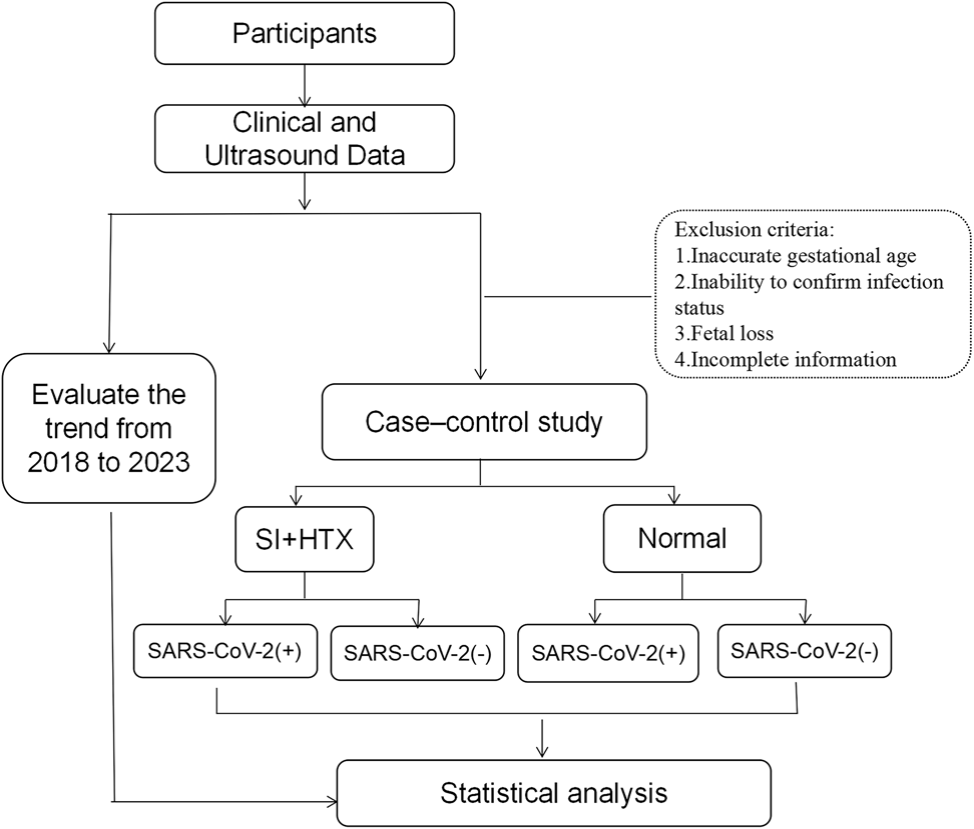
Research flowchart. SI, situs inversus; HTX, heterotaxy.

The flowchart was shown in Fig. 1. This study mainly included two parts, one was the fetal LR asymmetry disorders epidemic trend, and the other was the case–control study on LR asymmetry disorders and normal fetuses. Retrospective collection of detection data for fetal LR asymmetry disorders diagnosed from January 2018 to December 2023 to evaluate the trend in the incidence in recent years. Pregnant women were recruited from three medical centers. More than 10,000 newborns are born every year in every medical center. This retrospective multicenter observational study was approved by the Ethics Committee of our hospital (KYLL-202307-057). The research process and content have been explained to participants, and their informed consent has been obtained. All methods were performed in accordance with the relevant guidelines and regulations.

The case–control study part included fetuses with LR asymmetry disorders and normal fetuses in a 1:1 ratio, with a gestational age range of 14–39 weeks. Since the exposure factor was the SARS-CoV-2 infection that broke out at the end of 2022 in China, we chose fetuses with LR asymmetry disorders from January to May 2023 as the observation group. Each fetus was matched with a control group consisting of normal fetuses of similar gestational age, which were detected on the same day in the same medical center as the LR asymmetry disorders fetus. These control fetuses were included in the study. The study has specific exclusion criteria, which include: (1) inability to determine gestational age, (2) inability to confirm SARS-CoV-2 infection status, (3) fetal loss or death, (4) incomplete report data and low-quality stored images with obvious data errors, and (5) incomplete or unqualified questionnaire information.

The diagnosis of SARS-CoV-2 infection was confirmed based on clinical diagnostic criteria. These criteria include the presence of clinical manifestations related to SARS-CoV-2 infection and one or more positive results from etiological and serological tests. These tests include a positive SARS-CoV-2 nucleic acid test, a positive SARS-CoV-2 antigen test, a positive SARS-CoV-2 isolation and culture test, or a four times or more increase in SARS-CoV-2 specific immunoglobulin G (IgG) antibody levels in the recovery phase compared to the acute phase.

### Clinical and ultrasound variables

Clinical data collected through telephone inquiries and survey questionnaires primarily consist of personal information such as names, age, previous pregnancies, gestational age of confirmed LR asymmetry disorders, trimester of infection, vaccination completed, chromosome screening results, and history of LR asymmetry disorders. Additionally, the data includes information on SARS-CoV-2 infection and its associated symptoms such as fever, asthenia, dyspnea, cough, HNT (head, nose, throat) symptoms, headache, and myalgia. Furthermore, the ultrasound data provides specific information on LR asymmetry disorders and the presence of other deformities.

The interpretation of ultrasound reports was independently reviewed by two ultrasound physicians (Y.M.W. and Q.F.N.) with more than five years of experience. In case of any objections to the results, a physician (G.W.T.) with more than ten years’ experience will make the final determination. The process of obtaining case data involved a team of two researchers (H.F.W. and Q.L.) who worked together to retrieve, organize, and verify the data to ensure its reliability, completeness, and accuracy.

### Study size

This case–control study was conducted with two groups: the case group (N1) consisted of fetuses with LR symmetry disorders, and the control group (N2) consisted of normal fetuses. The main factor observed was SARS-CoV-2 infection. The matching principle used was that the case and control group had the same gestational age and were examined on the same day, at a matching ratio of 1:1. Based on current statistical data, the infection rate of SARS-CoV-2 in the observation group was approximately 96% at α = 0.05 and β = 0.1. The sample size formula was calculated, and it was found that N1 = N2 = 31 cases. Assuming a questionnaire pass rate of 90%, the required sample size is N1 = N2 = 35 cases.

### Statistical analysis

The statistical analyses were conducted using SPSS26.0 software. All tests were two-tailed, and a *p*-value of less than 0.05 was considered statistically significant. To assess the normality of the data distribution, we employed the Shapiro–Wilk test. For variables that exhibited a normal distribution, we reported the mean ± standard deviation. For variables that did not follow a normal distribution, we reported the median. We applied normal tests to continuous data, t-tests to normal data, and rank sum tests to skewed data. The classified data was analyzed using a chi-squared test.

## Results

### The incidence rate of LR asymmetry disorders

**Figure 2:**
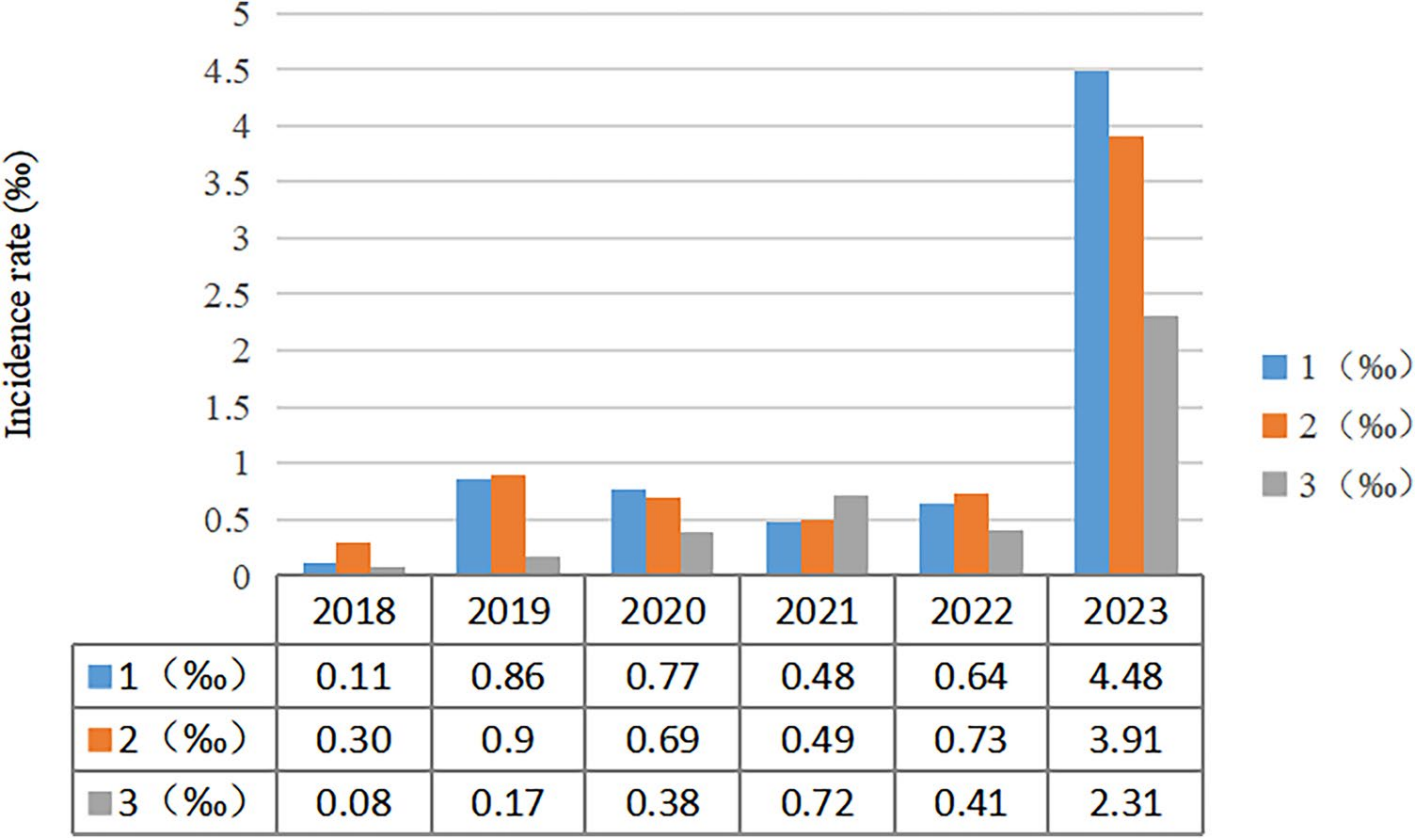
Incidence rate of fetal LR asymmetry disorders in three medical centers from 2018 to 2023.

**Figure 3:**
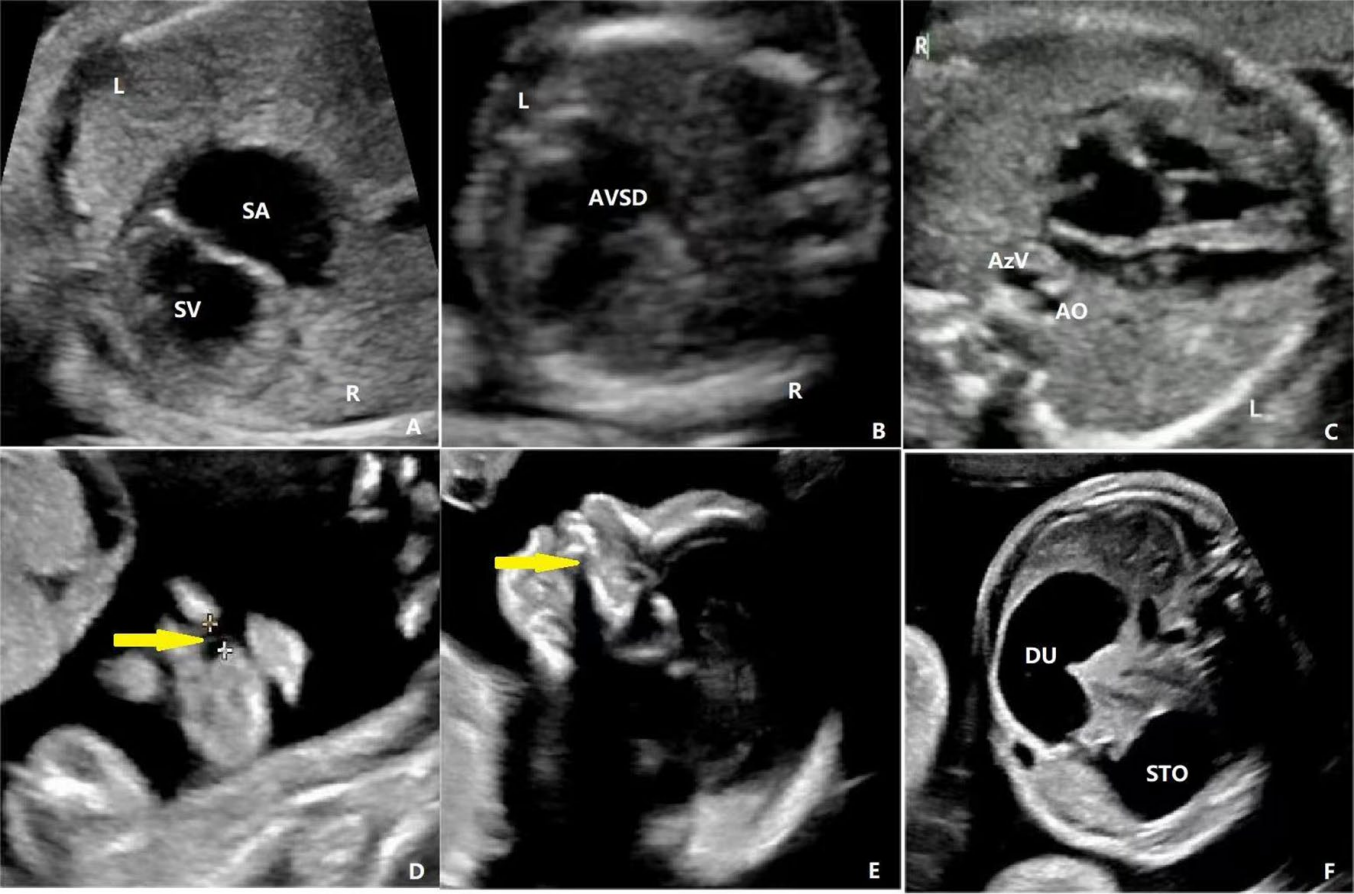
The features of LR asymmetry disorders in fetuses with cardiac and extracardiac abnormalities were as follows: (**a**) At 27 weeks, single ventricle was observed. The four chamber view showed only a single atrium and single ventricle. (**b**) At 16 weeks, SI was combined with atrioventricular septal defect at the four chamber view. Only one atrioventricular valve was observed. (**c**) At 26 weeks, there was left atrial isomerism with severance of the inferior vena cava and dilation of the azygos vein. (**d**) and (**e**) At 22 weeks, in the same pregnant woman, LR asymmetry disorders with cleft lip and cleft palate (yellow arrow) were observed. (**f**) At 24 weeks, LR asymmetry disorders were combined with gastrointestinal obstruction. SI, situs inversus; HTX, heterotaxy; LR, left–right; SA, single atrium; SV, single ventricle; AVSD, atrioventricular septal defect; AV, azygos vein; AO, aorta; DU, duodenum; ST, stomach; L, left; R, right.

From January 1, 2018 to December 31, 2023, a total of 151 cases of LR asymmetry disorders fetuses were confirmed across three medical centers, resulting in an incidence rate of approximately 0.85‰. The incidence rate from 2018 to 2023 was as follows: 0.17‰, 0.63‰, 0.61‰, 0.57‰, 0.59‰, and 3.24‰, respectively. Table 1 provided an annual breakdown of the number of detected cases of LR asymmetry disorders fetuses in each medical center from 2018 to 2023. Over the past six years, three medical centers have identified 55, 62, and 34 fetuses with LR asymmetry disorders, respectively. Additionally, Fig. 2 illustrated the incidence trend in these medical centers during the same period. The figure clearly demonstrated that the incidence rate remained consistently low between 2018 and 2022, ranging from 0.08 to 0.90‰. However, in the first half of 2023, there was a sudden and significant increase in the incidence rate, reaching a maximum of 4.48‰ (Fig. 3).

**Table 1:** The annual number of LR asymmetry disorders fetuses detected by three medical centers from 2018 to 2023.

	Medical Center 1(n)	Medical Center 2(n)	Medical Center 3(n)	Total
2018.1.1–2018.12.31	1	4	1	6
2019.1.1–2019.12.31	8	11	2	21
2020.1.1–2020.12.31	7	8	4	19
2021.1.1–2021.12.31	4	5	7	16
2022.1.1–2022.12.31	5	7	4	16
2023.1.1–2023.12.31	30	27	16	73
Total	55	62	34	151

LR, left–right.

### Participant characteristics in the control-study

A total of 49 mothers with LR asymmetry disorders fetuses were followed up for relevant information from January 1, 2023 to May 31, 2023. Among them, 5 cases were lost, 3 cases were uncertain whether they were infected with SARS-CoV-2, 6 cases were uncertain about the gestational age of infection, and 5 cases had no chromosome results. Finally, a total of 30 fetuses were included in the observation group. The confirmed minimum gestational age was 12 weeks, while the maximum gestational age was 28 weeks. Additionally, 30 normal fetuses were included in the control group in a 1:1 ratio. Please refer to Table 2 for more details.

**Table 2:** Baseline characteristics of pregnancies according to diagnosis of LR asymmetry disorders.

Characteristic	LR asymmetry disorders Fetuses (n = 30)	Normal Fetuses (n = 30)	*P*
Maternal Age	28.93 ± 4.91	30.97 ± 4.91	0.11
GA of confirmed LR asymmetry disorders	22.3 ± 4.2	22.33 ± 4.37	0.98
Previous pregnancies			0.19
None	16/30(53.33%)	12/30(40%)	
1–2	9/30(30%)	16/30(53.33%)	
3 +	5/30(16.67%)	2/30(6.67%)	
SARS-CoV-2			0.026*
Negative	1/30(3.33%)	8/30(26.67%)	
Positive	29/30(96.67%)	22/30(73.33%)	
Symptoms			
Fever	28/29(96.55%)	21/22(95.45%)	1.0
Asthenia	11/29(37.93%)	7/22(31.81%)	0.77
Dyspnea	0	0	-
Cough	20/29(68.97%)	9/22(40.91%)	0.05
HNT symptoms	17/29(58.62%)	11/22(50%)	0.58
Headache	12/29(41.38%)	5/22(22.73%)	0.23
Myalgia	17/29(58.62%)	10/22(45.45%)	0.41
Trimester of infection			0.008*
I	28/29(96.55%)	15/22(68.18%)	
II	1/29(3.45%)	3/22(13.64%)	
III	0	4/22(18.18%)	
Vaccination completed			0.68
None	6/30(20%)	4/30(13.33%)	
1	2/30(6.67%)	1/30(3.33%)	
2	3/30(10%)	6/30(20%)	
3	19/30(63.33%)	19/30(63.33%)	
Chromosome			1.0
Normal	29/30(96.67%)	30/30(100%)	
Abnormal	1/30(3.33%)	0	
Family History			–
Yes	0	0	
No	30/30(100%)	30/30(100%)	

LR, left–right ;GA, gestational age; HNT = head, nose, throat.

Significant values are in bold.

Among the 60 pregnant women included in this case–control study, a total of 51 were found to be infected with the SARS-CoV-2 virus, resulting in an infection rate of 85% (51/60). The most commonly reported symptom among the infected women was fever, observed in 96.08% (49/51) of cases, followed by cough, reported by 56.86% (29/51) of cases. The infection rate in the fetal LR asymmetry disorders group was found to be 96.67% (29/30), while in the control group it was 73.33% (22/30). This difference in infection rates between the two groups was statistically significant (*P* < 0.05). These findings suggest a significant association between SARS-CoV-2 infection and fetal LR asymmetry disorders.

It was found that 96.55% (28/29) of pregnant women with fetal LR asymmetry disorders were infected with SARS-CoV-2 in the first trimester, while 3.45% (1/29) were infected during the second to third trimester. The researchers utilized the Chi-squared test to analyze the gestational age of LR asymmetry disorders fetus infected with the SARS-CoV-2 virus. The results indicated significant differences in LR asymmetry disorders incidence among the different trimesters of infection (*P* < 0.05), suggesting a higher likelihood of fetal LR asymmetry disorders during the first trimester compared to other trimesters infected with SARS CoV-2(*P* < 0.05).

We conducted further analysis on LR asymmetry disorders fetuses infected with the SARS-CoV-2 virus in the first trimester. A total of 42 cases of fetuses with LR asymmetry disorders infected with the SARS-CoV-2 virus were identified. Among these cases, the majority of infections (88.1%, 37/42) occurred between 5 and6 weeks of gestation, while a smaller proportion (11.9%, 5/42) occurred between 7 and 8 weeks. Notably, no infections were observed between 9 and 12 weeks.

Out of 60 pregnant women, 83.33% (50/60) received the vaccine. The vaccination status can be categorized as follows: 5% (3/60) received one dose, 15% (9/60) received two doses, and 63.33% (38/60) received three doses. The study found no significant difference in the incidence of fetal LR asymmetry disorders among different vaccination states (*P* > 0.05). Additionally, the incidence of LR asymmetry disorders in non-vaccinated individuals did not show a significant difference compared to other vaccination states (*P* = 0.731, *P* > 0.05).

Our research revealed that whether SARS-CoV-2 infection occurred and the gestational age of infection were related to fetal LR asymmetry disorders occurred. Specifically, we observed that SARS CoV-2 infection and infection during the first trimester were identified as risk factors for fetal LR asymmetry disorders, with odds ratio (OR) values of 10.545 (95% CI 1.227, 90.662) and 13.067 (95% CI 1.467, 116.419) respectively. Furthermore, pregnant women infected during the first trimester had a higher probability of experiencing LR asymmetry disorders compared to those infected in the second and third trimesters.

### Associated malformations

A total of 151 cases of LR asymmetry disorders fetuses were identified from the databases of three medical centers, covering the period from 2018 to 2023. We conducted an analysis of ultrasound databases from three medical centers, examining whether fetuses with LR asymmetry disorders were associated with other malformations before and during the SARS CoV-2 pandemic (Table 3). A chi-square test was conducted to evaluate the occurrence of associated malformations in fetuses with LR asymmetry disorder before and during the SARS-CoV-2 pandemic. The test resulted in a p-value of 0.932, suggesting that there is no significant difference in the occurrence of associated malformations in LR asymmetry disorder fetuses before and during the pandemic.We found that 43.7% (66/151) of fetuses with LR asymmetry disorder had associated malformations. Among these, 90.9% (60/66) exhibited cardiac malformations, such as conotruncal anomalies, atrioventricular septal defects, single ventricle, and persistent left superior vena cava. Extra-cardiac malformations accounted for 19.7% (13/66), with the most common being upper digestive tract obstruction and cleft lip.

**Table 3:** Comparative analysis of Fetal LR asymmetry disorders during and prior to the SARS-CoV-2 pandemic.

	LR asymmetry disorders (n)		Total	*P*
	2018.1.1–2019.12.31	2020.1.1–2023.12.31		
Without associated malformation	15	70	85	0.932
Associated malformation	12	54	66	
Cardiac	11	42	53	
Extracardiac	1	5	6	
Cardiac and Extracardiac	0	7	7	
Total	27	124	151	

LR, left–right.

A detailed analysis was conducted to investigate the impact of SARS-CoV-2 infection on fetal left–right asymmetry disorder and associated malformations (Table 4). After excluding cases with loss to follow-up and those with uncertain diagnoses due to induced abortion, the study focused on the remaining 76 cases. Among the cases of LR asymmetry disorders, 65.79% (50/76) were without associated malformations, while 34.21% (26/76) had associated malformations. The most commonly observed associated malformation was cardiac malformation, accounting for 92.31% (24/26) of the cases.

**Table 4:** Associated malformation in pregnancies infected by SARS CoV-2 and non-infected pregnancies in LR asymmetry disorders fetuses.

	SARS CoV-2		Total	*P*
	Infected (n)	Non-infect (n)		
Without associated malformation	29	21	50	0.34
Associated malformation	12	14	26	
Cardiac	11	12	23	
Extracardiac	1	1	2	
Cardiac and Extracardiac	0	1	1	
Total	41	35	76	

LR, left–right.

## Discussions

Our study revealed a noteworthy correlation between SARS-CoV-2 infection during pregnancy and a substantial risk of fetal LR asymmetry disorders, particularly if the infection took place in the first trimester. Among the various malformations linked to fetal LR asymmetry disorders, cardiac malformation emerged as the most prevalent.

According to the previous report, the incidence of LR asymmetry disorders is approximately of 1:6000 to 1:10,000 live births^17,18^. Wang et al. investigated the incidence of fetal situs inversus in China over a period of ten years, from January 2014 to July 2023. The findings revealed that the incidence rate of fetal situs inversus in the first seven months of 2023 was more than four times higher than the average annual incidence rate observed from 2014 to 2022^19^. However, our findings indicated that in 2018 and 2019, before the SARS CoV-2 pandemic, the incidence of LR asymmetry disorders rate was 0.17‰ and 0.63‰, respectively, based on data retrieved from the databases of three medical centers. Despite the global spread of SARS-CoV-2 from 2020 to 2022, the number of people exposed to the virus in China is relatively low, resulting in a continued low prevalence at 0.61‰, 0.57‰, and 0.59‰, respectively. At the end of 2022, the outbreak of SARS CoV-2 increased population exposure to the virus.Subsequently, we found that the incidence of LR asymmetry disorders rapidly rose to 3.24‰, which is 6.3 times higher than the previous data. LR asymmetry disorders may vary depending on the virus strain. While LR asymmetry disorders is currently more prevalent during the Omicron dominance period, it remains uncertain whether other strains of the virus will also lead to LR asymmetry disorders in the Chinese population. This uncertainty arises from the limited exposure of the Chinese population to other virus strains.

In addition to our main objective of investigating potential influencing factors related to LR asymmetry disorders, our study also aimed to assess the associated risks through a case–control study. Visceral inversion arises from a defect in situs orientation, leading to various disturbances in laterality as a result of the inability to develop the typical LR asymmetry. The establishment of sidedness occurs early in development and is regulated by a cascade of signal molecules and genes, which undergo restriction in their expression during primitive streak formation (gastrulation)^20^. In our study, it was found that 96.55% of mothers with SARS-CoV-2 infection were infected during the first trimester, while 88.1% occurred between 5 and 6 weeks of pregnancy, which coincided with the formation of the gastrula.We hypothesize that during early pregnancy, particularly during the 5–6 week period of embryonic visceral development, SARS-CoV-2 interacts with the host chromatin, leading to alterations in gene expression and disease outcomes. The results of our study further support the notion that SARS-CoV-2 transmission during early pregnancy is associated with an increased risk of fetal LR asymmetry disorders.

According to the literature, the reported rate of fetal LR asymmetry disorders combined with various cardiovascular malformations ranged from 11 to 100%^21–23^. Cardiovascular malformations observed primarily include atrioventricular septal defect, atrioventricular arterial incoordination, Tetralogy of Fallot, single ventricle, double outlet of ventricle, and pulmonary artery abnormality^22,23^. Our study found that among LR asymmetry disorders fetuses, 90.9% were associated with cardiac malformations. The most common cardiac malformation observed was conotruncal anomalies, atrioventricular septal defects, single ventricle, and persistent left superior vena cava.These findings are consistent with the literature reports.

This study has several limitations that need to be acknowledged. Firstly, the data for this study was collected from only three medical centers, resulting in a relatively small sample size due to the extremely low incidence rate of LR asymmetry disorders. Additionally, it is important to note that this study was conducted during the SARS-CoV-2 outbreak in winter in China, and it remains uncertain whether infection with other viruses would have an impact on the results. Therefore, there is a possibility of selective bias and confounding bias. Secondly, due to the limitations of retrospective studies, some reports on visceral inversion fetuses lacked detailed information regarding the specific organs involved. As a result, it was challenging to determine whether the case was one of situs inversus or heterotaxy. Furthermore, due to the ongoing nature of this study, it is not feasible to track neonatal outcomes as many pregnant women have not yet given birth.

## Conclusion

SARS-CoV-2 infection during pregnancy is associated with a significant risk of fetal LR asymmetry disorders, especially when infection occurs in the first trimester. The most common type of malformation associated with fetal structural abnormalities is heart malformation. However, it is important to note that these results are preliminary and require careful interpretation before further information can be obtained. Regular ultrasound examinations of fetuses with structural abnormalities should be conducted on a monthly basis to assess their developmental status. We anticipate that postnatal follow-up of this group will provide a better understanding of the impact of prenatal SARS-CoV-2 infection.

## Data Availability

The datasets used and analysed during the current study are available from the corresponding author on reasonable request.

## References

[1] Soma-Pillay P, Nelson-Piercy C, Tolppanen H, Mebazaa A (2016). Physiological changes in pregnancy. Cardiovasc. J. Afr..

[2] Abu-Raya B, Michalski C, Sadarangani M, Lavoie PM (2020). Maternal immunological adaptation during normal pregnancy. Front. Immunol..

[3] Narang K, Enninga EAL, Gunaratne MDSK (2020). SARS-CoV-2 infection and COVID-19 during pregnancy: A multidisciplinary review. Mayo Clin Proc..

[4] Kucirka LM, Norton A, Sheffield JS (2020). Severity of COVID-19 in pregnancy: A review of current evidence. Am. J. Reprod. Immunol..

[5] Juan J, Gil MM, Rong Z, Zhang Y, Yang H, Poon LC (2020). Effect of coronavirus disease 2019 (COVID-19) on maternal, perinatal and neonatal outcome: systematic review. Ultrasound Obstet. Gynecol..

[6] Calvert C, Brockway MM, Zoega H (2023). Changes in preterm birth and stillbirth during COVID-19 lockdowns in 26 countries. Nat. Hum. Behav..

[7] McClymont E, Albert AY, Alton GD (2022). Association of SARS-CoV-2 infection during pregnancy with maternal and perinatal outcomes. JAMA..

[8] Arthurs AL, Jankovic-Karasoulos T, Roberts CT (2021). COVID-19 in pregnancy: What we know from the first year of the pandemic. Biochim Biophys. Acta Mol. Basis Dis..

[9] Wei SQ, Bilodeau-Bertrand M, Liu S, Auger N (2021). The impact of COVID-19 on pregnancy outcomes: A systematic review and meta-analysis. CMAJ.

[10] Narang K, Miller M, Trinidad C (2023). Impact of asymptomatic and mild COVID-19 infection on fetal growth during pregnancy. Eur. J. Obstet. Gynecol. Reprod. Biol..

[11] Soto-Torres E, Hernandez-Andrade E, Huntley E, Mendez-Figueroa H, Blackwell SC (2021). Ultrasound and Doppler findings in pregnant women with SARS-CoV-2 infection. Ultrasound Obstet. Gynecol..

[12] Ward JD, Cornaby C, Kato T (2022). The clinical impact of maternal COVID-19 on mothers, their infants, and placentas with an analysis of vertical transfer of maternal SARS-CoV-2-specific IgG antibodies. Placenta..

[13] Reppucci ML, Kaizer AM, Prendergast C (2023). Incidence of congenital complications related to COVID-19 infection during pregnancy. J. Neonatal Perinatal Med..

[14] Jacobs JP, Anderson RH, Weinberg PM (2007). The nomenclature, definition and classification of cardiac structures in the setting of heterotaxy. Cardiol. Young..

[15] Fabricius CO, Blalock A (1940). Situs inversus totalis and disease of biliary tract; survey of literature and report of case. Arch Surg..

[16] Vehsemeyer A. Ein fall von congenitaler Detiokardie: zugleich ein Beitrag zur Verwerthung der Röntgenstrahlen in Gebiete der inner Medizin [A case of congenital dextrocardia and a contribution to the utilization of X-rays in areas of internal medicine]. Deutsche Medizinische Wochenschrift. 1897;23(12):180–181. German.

[17] Peeters H, Devriendt K (2006). Human laterality disorders. Eur. J. Med. Genet..

[18] Lin AE, Ticho BS, Houde K, Westgate MN, Holmes LB (2000). Heterotaxy: Associated conditions and hospital-based prevalence in newborns. Genet. Med..

[19] Wang Y, Guo Z, Ye B (2023). Association of SARS-CoV-2 infection during early weeks of gestation with situs inversus. N. Engl. J. Med..

[20] Eitler K, Bibok A, Telkes G (2022). Situs inversus totalis: A clinical review. Int. J. Gen. Med..

[21] Walmsley R, Hishitani T, Sandor GG (2004). Diagnosis and outcome of dextrocardia diagnosed in the fetus. Am. J. Cardiol..

[22] Bernasconi A, Azancot A, Simpson JM, Jones A, Sharland GK (2005). Fetal dextrocardia: Diagnosis and outcome in two tertiary centres. Heart..

[23] Bohun CM, Potts JE, Casey BM, Sandor GG (2007). A population-based study of cardiac malformations and outcomes associated with dextrocardia. Am. J. Cardiol..

