# Author Correction: Increased risk of fetal left–right asymmetry disorders associated with maternal SARS-CoV-2 infection during the first trimester

**DOI:** 10.1038/s41598-024-67864-3

**Published:** 2024-07-18

**Authors:** Yang Li, Yuemei Wang, Haifang Wu, Qi Li, Shizhen Li, Chunli Qiu, Shuo Qiu, Qingfang Niu, Xianmei Zhang, Yi Xiong, Guowei Tao

**Affiliations:** 1 Department of Medical Ultrasound, The First Affiliated Hospital of Shandong First Medical University and Shandong Provincial Qianfoshan Hospital, Shandong Medicine and Health Key Laboratory of Abdominal Medical Imaging, Jinan, China; 2 The Second Affiliated Hospital of Shandong First Medical University, Taian, 271000 China; 3 Jinan Maternal and Child Health Care Hospital, Jinan, China; 4 Qilu Hospital of Shandong University, No. 107, Wenhua West Road, Jinan City, 250012 China; 5 Linyi Maternal and Child Health Care Hospital, Shenzhen, China; 6 Luohu People’s Hospital, Third Affiliated Hospital of Shenzhen University, Shenzhen, China

Correction to: *Scientific Reports* 10.1038/s41598-024-61778-w, published online 19 May 2024

The original version of this Article contained an error in Affiliation 1, which was incorrectly given as ‘The First Affiliated Hospital of Shandong First Medical, Jinan, China’. The correct affiliation is listed below:

Department of Medical Ultrasound, The First Affiliated Hospital of Shandong First Medical University & Shandong Provincial Qianfoshan Hospital, Shandong Medicine and Health Key Laboratory of Abdominal Medical Imaging, Jinan, China

The original Article has been corrected.

